# Hidden in Plain Sight: Antisynthetase Syndrome Masquerading As Acute Coronary Syndrome and Heart Failure

**DOI:** 10.7759/cureus.74169

**Published:** 2024-11-21

**Authors:** Ahmed Fadel, Rekha Bhalla, Sarmad Mushtaq

**Affiliations:** 1 Respiratory Medicine, Dartford and Gravesham National Health Service (NHS) Trust, Dartford, GBR

**Keywords:** anti-jo-1 antibodies, antisynthetase syndrome, connective tissue disease associated interstitial lung disease, high-dose methylprednisolone, s: dermatomyositis, tacrolimus

## Abstract

A middle-aged man presented with fever, generalized myalgia, dyspnea, and worsening chest discomfort. Initial investigations showed elevated troponin and creatinine kinase levels and nonspecific ECG changes. Pulmonary embolism was ruled out, and high-resolution CT (HRCT) revealed ground-glass opacities and nonspecific interstitial pneumonia (NSIP)/organizing pneumonia (OP) overlap pattern. Given the elevated creatinine kinase level, antisynthetase syndrome with rapidly progressive interstitial lung disease (RP-ILD) was suspected. Treatment with pulse-dose methylprednisolone and tacrolimus led to significant clinical improvement, with HRCT showing resolution of the NSIP/OP findings on follow-up. This case highlights the importance of early diagnosis and multidisciplinary management in antisynthetase syndrome.

## Introduction

Antisynthetase syndrome (ASSD) is a rare systemic autoimmune disorder characterized by the presence of autoantibodies directed against aminoacyl-tRNA synthetases, which are enzymes critical for protein synthesis. It is a subset of idiopathic inflammatory myopathies (IIM) that primarily involves interstitial lung disease (ILD), myositis, and arthritis. The classic clinical triad of ILD, myositis, and arthritis is seen in approximately 90% of patients, although not always simultaneously. Other notable features include Raynaud’s phenomenon, fever, and mechanic hands. The pulmonary component, particularly ILD, is a significant cause of morbidity and mortality in ASSD and often dictates patient prognosis. Due to its heterogeneous presentation and overlap with other connective tissue diseases (CTDs), diagnosing ASSD can be challenging, especially when extramuscular manifestations such as ILD precede or occur in the absence of muscle involvement. The presence of antisynthetase antibodies such as anti-Jo-1, anti-PL7, and anti-PL12, plays a crucial role in diagnosis [1]. These antibodies are often associated with a more severe disease course, including rapidly progressive ILD (RP-ILD). This case report presents a patient with ASSD manifesting as RP-ILD, who showed significant improvement following prompt initiation of corticosteroids and tacrolimus. This case underscores the importance of early recognition and the use of a multidisciplinary approach in managing ASSD to improve outcomes.

## Case presentation

**Figure 1:**
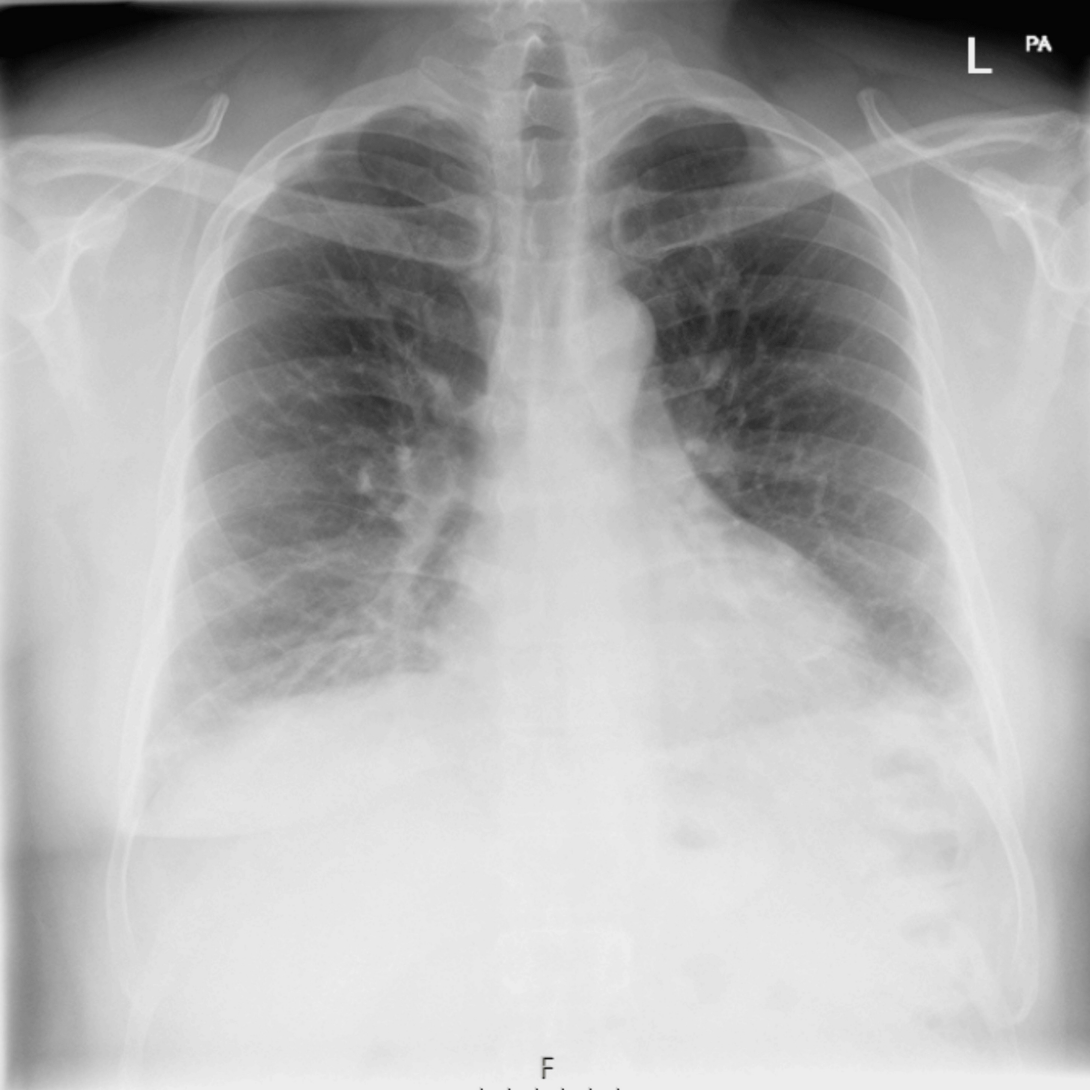
Chest X-ray: Bilateral basal consolidations, and reticular changes.

A 46-year-old man with no significant medical history or recent travel presented to the emergency department with fever, generalized myalgia, dyspnea, and cough. Clinical examination revealed bilateral basal crackles; however, no rash, Raynaud’s phenomenon, or eruptions on the fingers were observed. Initial blood investigations showed normal levels of inflammatory markers, including a CRP of 4.1 mg/L (reference range <5 mg/L) and a white blood cell count of 6 × 10^9/L (reference range 4-11 × 10^9/L). Mildly elevated troponin levels were detected at 23.5 ng/L and 25.5 ng/L (reference range <14 ng/L), though the patient did not report chest pain, and the ECG showed no acute ischemic changes. Chest radiography revealed bilateral basal consolidation and reticular changes (Figure 1). 

**Figure 2:**
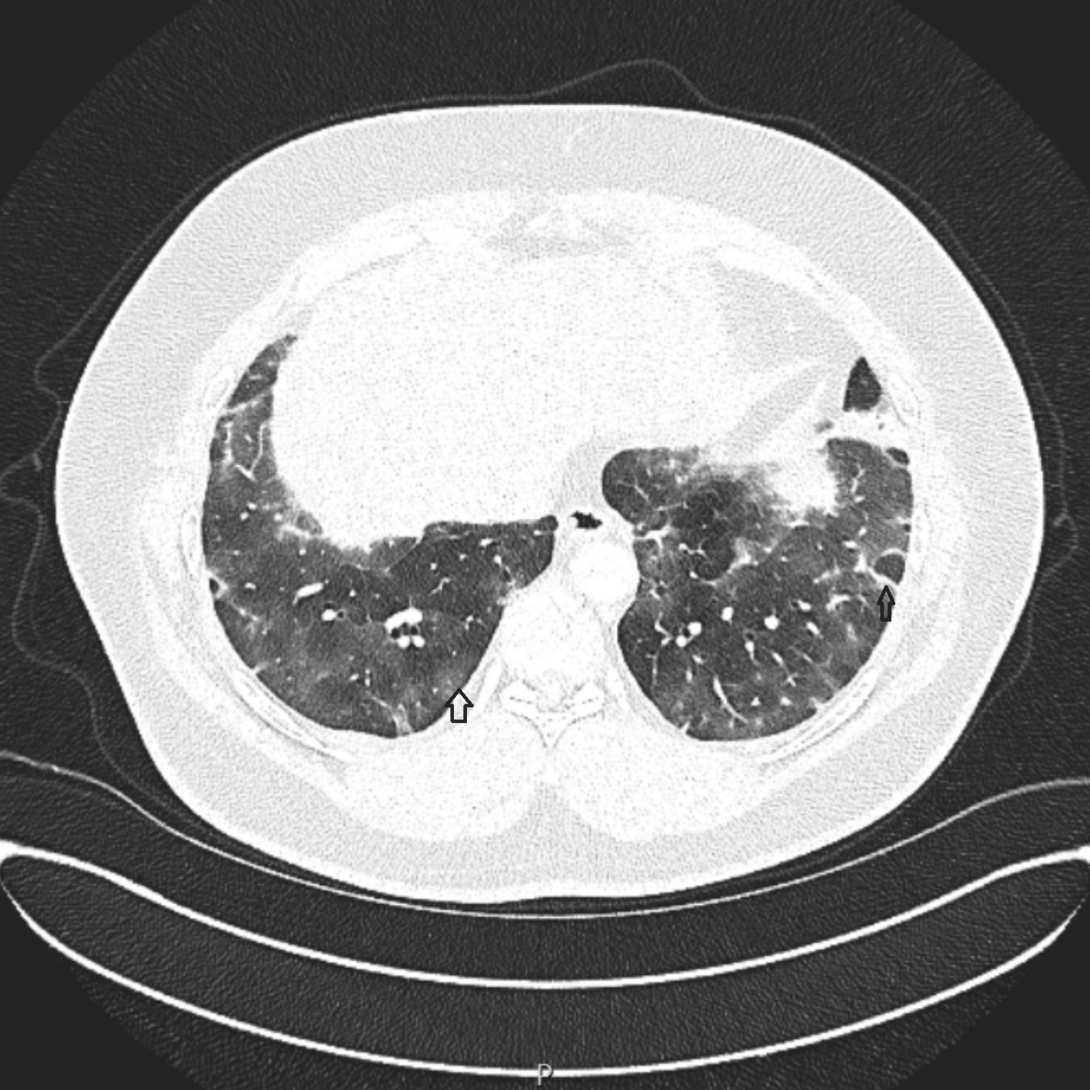
Bilateral ground glass changes, predominantly basal, with perilobular distribution.

Given the elevated troponin levels and dyspnea, pulmonary embolism was considered and a CT pulmonary angiography (CTPA) was performed. CTPA ruled out pulmonary embolism but revealed bilateral ground-glass opacities, predominantly in the basal regions. Despite negative COVID-19 test results, bronchoscopy was not performed at this stage to confirm the diagnosis or exclude other atypical infections. The patient was clinically diagnosed with COVID-19 on the basis of his symptoms. As he remained clinically stable with normal oxygen requirements, he was discharged with instructions to follow up if the symptoms did not resolve or worsen (Figure 2).

However, the patient’s condition did not improve. He experienced progressive shortness of breath, worsening myalgia, proximal muscle weakness, and symmetrical arthritis with swelling in the bilateral metacarpophalangeal and interphalangeal joints. Six weeks later, the patient was readmitted due to increased oxygen requirements and worsening chest discomfort. Blood tests revealed elevated troponin (105.6 ng/L, reference range <14 ng/L), CRP (16.9 mg/L, reference range <5 mg/L), and creatinine kinase (2488 U/L, reference range 30-200 U/L) levels, along with nonspecific ECG changes. Non-ST-elevation myocardial infarction (NSTEMI) was suspected, and coronary angiography revealed patent coronary arteries with no occlusion.

**Figure 3:**
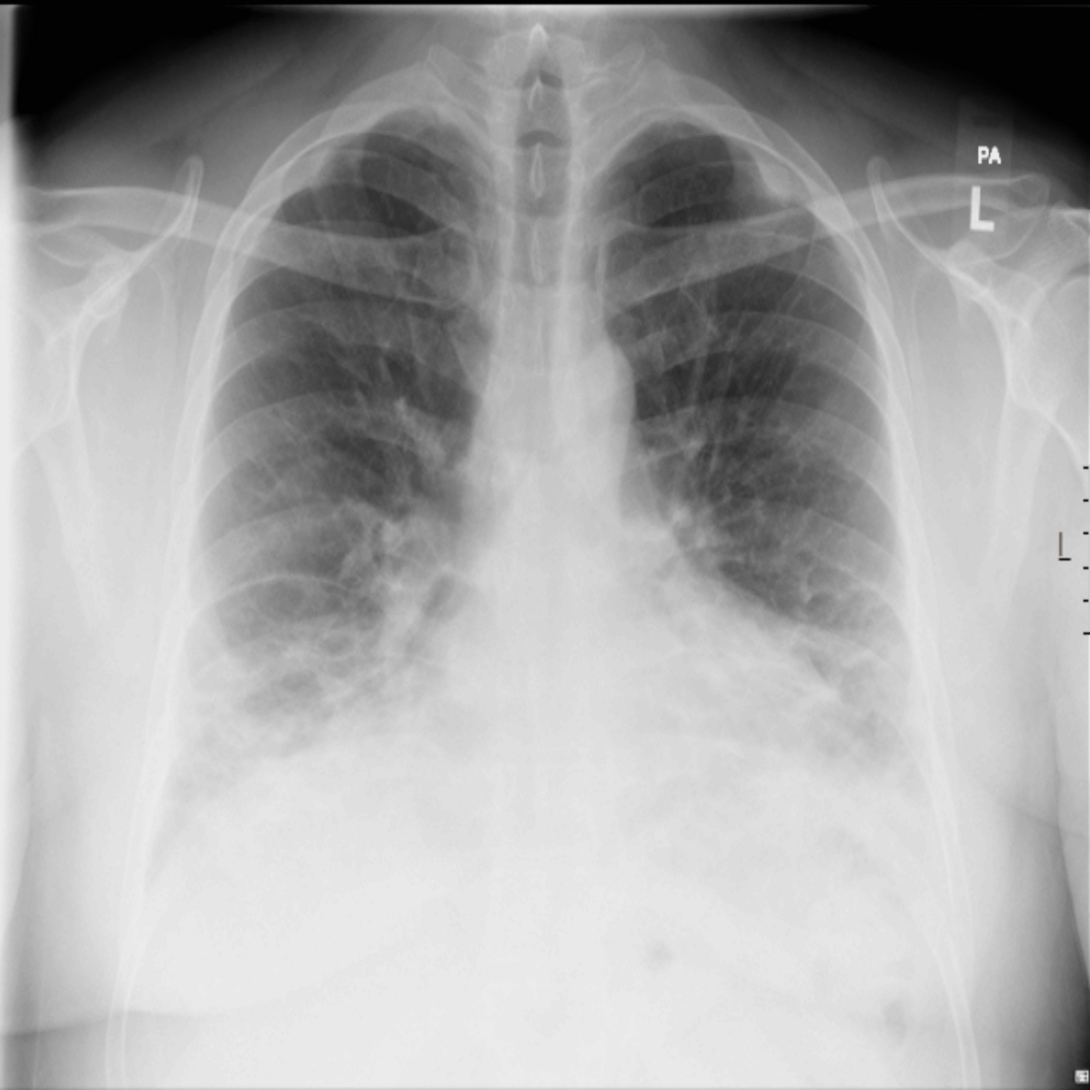
Bilateral predominately basal consolidative changes.

Repeat chest radiography revealed increased bilateral consolidations, predominantly in the basal regions (Figure 3).

**Figure 4:**
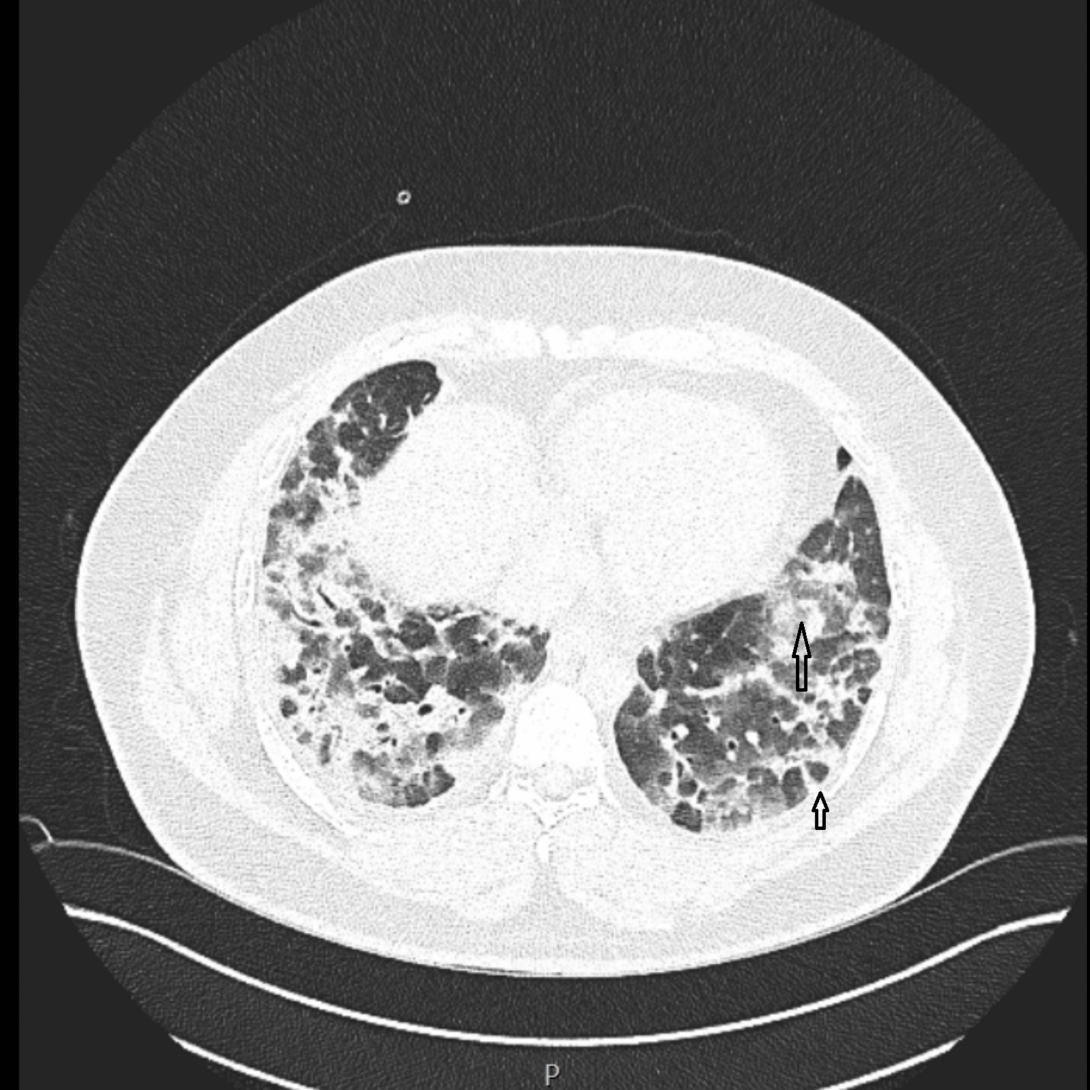
Bilateral ground-glass opacities, traction bronchiectasis, along with bilateral basal consolidation in a perilobular distribution consistent with organizing pneumonia.

A V/Q scan did not reveal pulmonary embolism. However, high-resolution CT (HRCT) of the chest revealed extensive bilateral ground-glass opacities consistent with nonspecific interstitial pneumonia (NSIP) pattern, as well as consolidative changes with a perilobular distribution, suggestive of organizing pneumonia (OP). Early traction bronchiectasis was observed in the middle and lower lobes (Figure 4). These findings raised the suspicion of CTD-ILD, particularly idiopathic inflammatory myopathy (IIM) with ASSD, due to elevated creatinine kinase levels, which indicated concurrent myositis.

A thorough history and physical examination confirmed generalized myalgia and proximal muscle weakness but no signs of mechanic’s hands or Raynaud’s phenomenon.

An extensive autoimmune and infectious workup was conducted, including testing for ENA, ANCA, and a myositis panel, along with screening for *Pneumocystis jirovecii* pneumonia (PJP), fungal infections, and an extended viral panel (Table 1). The patient tested positive for anti-Jo-1 antibodies, anti-RO-52, and PL-12 antibodies, confirming the diagnosis of ASSD. These findings, along with the imaging results, supported the clinical suspicion of rapidly progressive interstitial lung disease (RP-ILD).

**Table 1:** Extended autoimmune and infectious causes workup PJP: *Pneumocystis jirovecii* pneumonia; CMV: cytomegalovirus; EBV: Epstein-Barr virus

Test	Result	Reference Range
ACE level	46 U/L	20-70 U/L
ANCA pattern	Negative	Negative
Anti-nuclear Ab	Negative	Negative
Anti-RNP antibodies	Negative	Negative
La (SSB) antibodies	Negative	Negative
Sm antibodies	Negative	Negative
Ro (SSA) antibodies (Ro60 antibody)	Negative	Negative
Ro52 antibody	Positive	Negative
Jo-1 antibodies	Positive	Negative
PL-12 antibody	Positive	Negative
PL-7, SRP, Ku, Mi-2, PM-Scl75, PM-Scl100, TIF1 gamma, MDA5, NXP2, SAE1, OJ, EJ	Negative	Negative
Scl-70 antibodies	Negative	Negative
dsDNA Ab	Negative	Negative
Rheumatoid factor	1 IU/mL	<14 IU/mL
Anti-CCP antibody	Negative	<20 U/mL
PJP PCR	Negative	Negative
Extended viral screen (Adenovirus, CMV, EBV)	Negative	Negative
HIV	Negative	Negative
B-D-glucan, galactomannan	Negative	Negative

The patient was treated with pulse-dose intravenous methylprednisolone at 1 g daily for three days, alongside broad-spectrum antibiotics to cover potential infectious causes, including PJP, which was later ruled out. Steroids were then tapered to oral prednisolone at a dose of 1 mg/kg. The patient experienced dramatic improvement, with oxygen requirements progressively reduced until oxygen support was no longer necessary. Complete resolution of the arthritis and myositis was also observed.

A multidisciplinary discussion was held at a tertiary center involving both respiratory and rheumatology teams. The plan was to introduce a steroid-sparing agent, with consideration of azathioprine or mycophenolate mofetil. Azathioprine was started because of associated myositis [2]. However, testing for thiopurine methyltransferase (TPMT) showed a level of 25 pmol/h/mg Hb, indicating that the patient was a carrier of TPMT deficiency. Given the rapidly progressive ILD and elevated troponin levels suggesting myocarditis, tacrolimus was initiated as an add-on therapy. Cyclophosphamide and rituximab were not considered as they are typically used as rescue therapy for steroid-refractory rapidly progressive ILD [2]. The prednisolone dose was gradually reduced and the patient was maintained at 10 mg daily. Tacrolimus was increased to 3 mg twice daily, with adjustments based on therapeutic levels.

**Figure 5:**
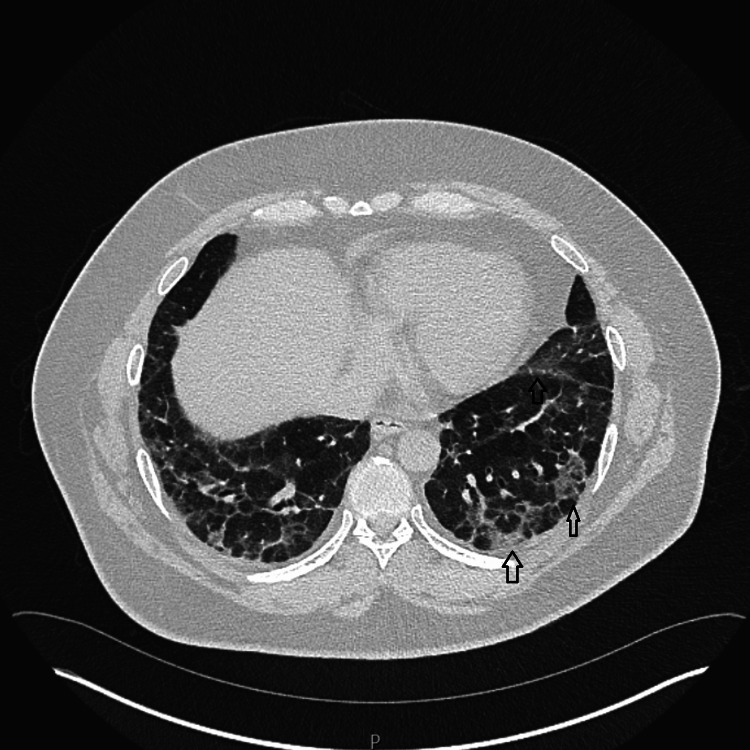
Follow-up HRCT chest: Improvement of ground-glass opacities, consolidations, and traction bronchiectasis. HRCT: high-resolution CT

Follow-up HRCT of the chest (Figure 5), performed two months after discharge showed significant improvement in the NSIP/OP overlap pattern, correlating with the patient’s clinical recovery.

A cardiac MRI was performed to check for ongoing myocarditis, which was suspected during the patient’s initial presentation owing to elevated troponin levels. However, no manifestations of myocarditis were observed.

## Discussion

ASSD is a subset of IIM. It is primarily characterized by the presence of antisynthetase antibodies, ILD, myositis, and arthritis. However, these clinical features may not always appear simultaneously, which makes early diagnosis challenging. Morbidity and mortality in ASSD are predominantly related to pulmonary system involvement. ASSD is a rare and heterogeneous systemic autoimmune disease (SAID) characterized by the presence of anti-aminoacyl-tRNA synthetase (anti-ARS) autoantibodies, which include anti-Jo-1, anti-PL-7, anti-PL-12, anti-EJ, anti-OJ, anti-KS, anti-Zo, and anti-YRS/HA [3].

Although there is a lack of consensus-driven criteria for the definition of ASSD, expert opinion-driven criteria were used. This includes the presence of ARS autoantibodies and one feature of the classical clinical triad (arthritis, myositis, or ILD) [4].

Connors criteria for diagnosis of ASSD [2]

Anti-ARS antibody plus one of the following: ILD by American Thoracic Society (ATS) criteria; arthritis (clinical, radiological, self-report); unexplained fever; Raynaud’s phenomenon; mechanic’s hand

Epidemiology

The first description of ASSD was by Hochberg et al., who noted a high prevalence of anti-Jo-1 antibodies in polymyositis or polymyositis overlap syndromes associated with ILD. The global prevalence of ASSD is 1-9 per 100,000 people, according to Orphanet data, with antisynthetase antibodies present in 11.1-39.19% of patients with IIM [5]. ASSD is 2-3 times more common in women than in men, with the onset age in adults ranging from 19 to 82 years, and is much rarer in children and adolescents [5].

Clinical Presentation

The clinical manifestations of ASSD are diverse, with the classic triad of arthritis, myositis, and ILD observed in up to 90% of the cases. However, only 20% of patients initially present with the complete triad, according to the American and European Network of Antisynthetase Syndrome (AENEAS). Other components often develop throughout the disease course. Raynaud’s phenomenon, fever, and mechanic’s hands may also occur. Approximately 25% of ASSD patients present with isolated arthritis, with 70% showing a symmetrical distribution resembling rheumatoid arthritis (RA), leading to potential misclassification, especially in anti-CCP or RF-positive patients. Anti-CCP positivity was associated with more severe erosive arthritis in patients with ASSD. Monitoring myositis and ILD in arthritis-only presentations is crucial. The prevalence of myositis at presentation varied, ranging from 23.5% in the Pittsburgh cohort to 52% in the AENEAS cohort. It is more common in anti-Jo-1, anti-EJ, and anti-PL-7-positive patients, while anti-PL-12, anti-KS, and anti-OJ patients often follow an amyopathic course [1].

Myocarditis in ASSD is underrecognized; in a study conducted on 352 ASSD patients, the prevalence of myocarditis was found to be 3.4% [5]. Myocarditis presents a spectrum of clinical manifestations, ranging from asymptomatic cases to heart failure and arrhythmias, with dyspnea being the most frequent [6].

The diagnosis of myocarditis can be challenging because of its subclinical and nonspecific presentation. Elevated troponin I/T and cardiac MRI findings are crucial diagnostic tools for increased extracellular volume (ECV), late gadolinium enhancement (LGE), and increased T1 and T2 mapping, which are indicative of myocardial inflammation. The use of the 2018 Lake Louise Criteria, incorporating these advanced techniques, has demonstrated increased sensitivity for diagnosing myocarditis compared with earlier methods [7].

Pulmonary involvement, predominantly ILD, is the leading cause of morbidity and mortality in patients with ASSD. The prevalence of ILD in patients with ASSD ranges from 67% to 100%, making it more common than non-ASSD-related IIM, where ILD occurs in 20% to 75% of cases. Key predictors of progressive lung disease in ASSD include the presence of non-Jo-1 autoantibodies such as anti-PL7 and anti-PL12, male sex, older age, low diffusion capacity (DLCO) at diagnosis, and the co-occurrence of anti-Ro52 antibodies. In a study of 36 patients, middle-lobe traction bronchiectasis was associated with poorer long-term prognosis, whereas its absence was linked to better long-term prognosis [8].

The radiologic presentation of ILD in ASSD often shows a pattern of nonspecific interstitial pneumonia (NSIP) followed by OP. NSIP and OP overlap is notably associated with CTD-ILD, specifically IIM [9]. This overlap is associated with a more unfavorable prognosis [10].

Pulmonary arterial hypertension (PAH) is a significant, but often overlooked, complication in ASSD. In the Pittsburgh myositis cohort, PAH was present in 11% of anti-Jo-1-positive patients and 21% of non-Jo-1 ASSD patients. PAH was the second leading cause of death, accounting for 11% of the mortalities. A French study reported PAH in 25% of patients with ASSD, but only 8% had severe cases confirmed by catheterization. Many cases may go unreported because of limited testing. PAH in ASSD patients has a poor prognosis, with a three-year survival rate of 58%. Regular screening via echocardiograms is advised to detect early signs, such as exercise-induced desaturation or dyspnea. Dermatological features like “Hiker’s feet” and mechanic’s hands are associated with an increased risk of ILD in ASSD, though these can also occur in other idiopathic inflammatory myopathies (IIM)[1].

Pathogenesis

Antisynthetase antibodies (ARS) are key to diagnosing and understanding ASSD. These antibodies target aminoacyl-tRNA synthetases that are crucial for protein synthesis. Among ARS, anti-Jo-1 is the most common, found in 20-30% of patients with IIM, whereas anti-PL-7 and anti-PL-12 are rare. ARS antibodies, particularly anti-Jo-1, trigger immune responses that lead to inflammation and tissue damage, particularly in the lungs and muscles. Environmental factors such as dust, gases, or smoke can trigger ASSD in genetically predisposed individuals, with smoking linked to a higher risk of anti-Jo-1 antibodies. Genetic markers, such as HLA-B08:01 and HLA-DRB103:01, also increase susceptibility. Together, these genetic and environmental factors drive disease onset. ARS antibodies not only help diagnose ASSD but also play a central role in disease progression, making them critical for managing inflammation and lung damage [5].

The overlap between IIM and other CTDs is well-documented. Antibodies, such as anti-PM/Scl, anti-Ku, and anti-U1 RNP, frequently occur in these overlapping syndromes. Studies show that 6.5%-36.7% of IIM patients have CTD overlap, most commonly with systemic sclerosis (SSc). Scleromyositis, which combines features of both SSc and myositis, appears in 30%-50% of cases [1].

The link between dermatomyositis (DM) and malignancy is well recognized, with standardized incidence ratios (SIRs) ranging from 3.0 to 6.0. Common cancers include ovarian, lung, breast, and gastrointestinal. Risk factors include older age, rapid onset, and weight loss. Conversely, ILD, arthritis, and Raynaud’s phenomenon lower malignancy risk. ILD appears in 5-40% of DM cases and, when combined with malignancy, can lead to severe disease. Although malignancy is less common in ASSD, antisynthetase antibodies, such as anti-Jo-1, do not exclude cancer risk. Antibodies like anti-PL-12 and anti-PL-7 are linked to ILD, while myositis-specific antibodies (MSAs) like anti-TIF1-γ or anti-NXP-2 suggest a higher malignancy risk [11].

Treatment

Corticosteroids

These are the mainstays of initial therapy and are often started at high doses (e.g., prednisone 1 mg/kg/day) to control both lung and muscle diseases. However, corticosteroids alone are frequently insufficient for long-term disease control and are associated with significant side effects, necessitating the addition of steroid-sparing agents.

Immunosuppressive Agents

Azathioprine and Mycophenolate Mofetil: These are common second-line agents that are used in combination with corticosteroids. Azathioprine is effective in managing muscle symptoms, whereas mycophenolate mofetil is often preferred for ILD because of its tolerability.

-Tacrolimus: A calcineurin inhibitor that is increasingly used in refractory cases, especially when ILD is present. It has been shown to improve lung function and muscle strength while allowing for steroid tapering.

-Cyclophosphamide: This is reserved for severe, rapidly progressing ILD or refractory diseases. While effective, it is associated with significant toxicity, including an increased risk of malignancy, sterility, and cytopenia.

- Rituximab: Rituximab, a B-cell depleting agent, is used in severe or refractory cases, particularly in patients with progressive ILD who fail to respond to standard immunosuppressive therapy [2].

-Janus kinase inhibition (JAKi) therapy in refractory ASSD in a retrospective study [11].

-Tocilizumab is used in refractory ASSD as evidenced in case reports [12].

-Lung transplant should be considered for patients with advanced ILD whose clinical condition deteriorates despite maximal medical therapy, providing a definitive option for those with treatment-refractory disease [13].

The link between dermatomyositis (DM) and malignancy is well recognized, with SIRs ranging from 3.0 to 6.0. Common cancers include ovarian, lung, breast, and gastrointestinal. Risk factors include older age, rapid onset, and weight loss. Conversely, ILD, arthritis, and Raynaud’s phenomenon lower malignancy risk. ILD appears in 5-40% of DM cases and, when combined with malignancy, can lead to severe disease. Although malignancy is less common in ASSD, antisynthetase antibodies, such as anti-Jo-1, do not exclude cancer risk. Antibodies like anti-PL-12 and anti-PL-7 are linked to ILD, while MSAs like anti-TIF1-γ or anti-NXP-2 suggest a higher malignancy risk [14].

A multidisciplinary approach is essential for diagnosing and managing ASSD, as it affects multiple systems, particularly muscles, lungs, and joints. Coordination among rheumatologists, pulmonologists, radiologists, and other specialists is key. Respiratory involvement, especially ILD, is managed by respiratory specialists, whereas rheumatologists focus on muscle and joint inflammation. Radiologists play a critical role in interpreting high-resolution computed tomography (HRCT) scans, which are crucial in diagnosing ILD. Immunologists help diagnose ASSD by identifying antibodies, such as anti-Jo-1. This collaborative, holistic approach ensures accurate diagnosis, ongoing monitoring, and treatment adjustments, which are vital for managing the complex features of ASSD and improving patient outcomes [15].

## Conclusions

ASSD is a rare autoimmune disease characterized by the involvement of the lungs, muscles, and joints, with ILD being the primary cause of morbidity and mortality. Diagnosis can be challenging owing to its variable clinical presentation, but the presence of anti-aminoacyl-tRNA synthetase antibodies, especially anti-Jo-1 antibodies, is a key diagnostic marker. Although there are no universal diagnostic criteria, the combination of these antibodies with myositis, arthritis, or ILD typically guides diagnosis.

A multidisciplinary approach involving pulmonologists, rheumatologists, and radiologists is essential for managing ASSD. Initial treatment usually involves corticosteroids, and in more resistant cases, immunosuppressive agents or biologics, such as rituximab, may be used. Early detection is critical for improving patient outcomes. Unfortunately, in this case, ASSD was not initially considered, which led to readmission due to respiratory failure.

Management of complications such as PAH and myocarditis is crucial for improving outcomes. Although malignancy is less common in ASSD than in dermatomyositis, certain antibodies and overlapping syndromes may increase the risk of cancer, necessitating a thorough workup. Overall, the prognosis depends on early diagnosis, aggressive pulmonary management, and coordinated multidisciplinary care.
